# Obstetrical and neonatal case definitions for immunization safety data

**DOI:** 10.1016/j.vaccine.2016.08.026

**Published:** 2016-12-01

**Authors:** Robert T. Chen, Pedro L. Moro, Jorgen Bauwens, Jan Bonhoeffer

**Affiliations:** Division of HIV/AIDS Prevention, National Center for HIV/AIDS, Viral Hepatitis, STD, and TB Prevention, CDC, USA; Immunization Safety Office, Division of Healthcare Quality Promotion, Centers for Disease Control and Prevention (CDC), Atlanta, GA, USA; Brighton Collaboration Foundation, Spitalstrasse 33, 4056 Basel, Switzerland

Administering vaccines specifically to pregnant women as part of a disease prevention strategy has not been routinely practiced, with the major exception of protecting against neonatal tetanus. Pregnancy continues to be a contraindication for receipt of most live viral (e.g., measles, mumps, rubella, varicella) vaccines. In the wake of the thalidomide tragedy [1], real or perceived risks of teratogenicity have served as a deterrent to administration of many medicines during pregnancy. Little is known about the effects of new medications or vaccines on pregnant women since they are often excluded from clinical research designed to determine safety. In recent years, however, a groundswell of public and provider opinion has developed urging reconsideration of the practice of excluding pregnant women from drug safety studies for some vaccines. The morbidity and mortality following some infections (e.g., influenza and pertussis) can be disproportionally greater among infants and pregnant women [2], [3]. Consequently, the Advisory Committee on Immunization Practices (ACIP) has recommended routine immunization of pregnant women with influenza vaccine since 1997 [2] and a dose of Tdap during every pregnancy since 2012 [4]. Future vaccines for use in pregnancy may include group B streptococcal, Zika, and respiratory syncytial virus vaccines. The challenges and opportunities for maternal immunization have begun to be addressed in various vaccine stakeholder meetings. For example, the US National Vaccine Advisory Committee (NVAC formed a Maternal Immunization Working Group in 2012 and issued a report in 2014 on “Reducing Patient and Provider Barriers to Maternal Immunizations” [6].

Post-marketing surveillance of adverse events in pregnant women is warranted, not only because of the lack of pre-licensure vaccine safety data in pregnancy, but also to assess for rarer adverse events in pregnant women or their infants. Unlike efficacy or effectiveness, however, safety cannot be assessed directly. The relative safety of a vaccine can only be inferred indirectly from the absence of multiple possible adverse events following immunizations (AEFI) that have been assessed in a relatively large population. Historically, there was relatively little standardization of case definitions for AEFI [7]. This resulted in a huge missed opportunity for meaningful comparison of safety data across vaccine trials and studies in pre- and post-licensure settings, which in turn hindered our ability to advance the science of vaccine safety.

The Brighton Collaboration was formed in 2000 to help overcome these shortcomings [7]. Over 30 standardized case definitions (with accompanying guidelines on data collection) have since been developed by global experts for use in various settings, arrayed by the level of available evidence. The Brighton Collaboration case definitions are recommended for use by normative bodies such as the FDA, the European Medicines Agency, the US Centers for Disease Control and Prevention (CDC), and the European Centre for Disease Prevention and Control [8].

Recent studies of Guillain-Barré syndrome (GBS) [9], [10], [11] and narcolepsy [12], [13], [14] after influenza vaccination in multiple countries used Brighton case definitions for these conditions, thereby increasing their scientific comparability. Brighton case definitions can also be important during public health emergencies as exemplified by the recent development of a microcephaly definition (and use of the GBS case definition) [15] in response to the Zika outbreak in the Americas [16].

Pregnant women and their infants constitute a special population group where safety monitoring for adverse events is becoming an important activity as new vaccines are being developed and become recommended for use in the near future [5]. The development of standard definitions for pregnant women and infant outcomes would be a great benefit to research efforts aimed at monitoring vaccine safety in these populations. Accordingly, the World Health Organization (WHO) and Brighton Collaboration jointly convened a meeting in July 2014 to develop Brighton Collaboration definitions of key terms used for monitoring the safety of immunization in pregnant women and infants [17]. As described by Bonhoeffer et al. [18] in this special issue, this 2-day meeting led to the formation of the Global Alignment of Immunization safety Assessment in pregnancy (GAIA) project funded by the Bill and Melinda Gates Foundation. This special issue of Vaccine includes the hard work by members of the GAIA project working groups with the development of five obstetric case definitions (Non-reassuring fetal status, Maternal death, Postpartum hemorrhage, Hypertensive disorders of pregnancy, and Preterm birth) and five neonatal case definitions (Congenital anomalies, Neonatal death, Neonatal infections, Preterm birth, Stillbirth) in the first year. Meanwhile, the next ten GAIA case definitions are under development, including: (1) Obstetric: Abortion, Antenatal bleeding, Gestational diabetes, Dysfunctional labor, Fetal growth retardation; and, (2) Neonatal: Low birth weight, Small for gestational age, Neonatal encephalopathy, Respiratory distress, Failure to thrive. These additional GAIA case definitions will be published together in a future special issue of Vaccine when ready.

The more than 200 volunteers from 13 organizations who formed the ten case definition working groups are to be congratulated for their willingness to cross their traditional “silos” (e.g., vaccines, obstetrics, neonatology) in this large collaboration. In addition to the main case definitions themselves, the GAIA collaboration has developed several associated tools to facilitate their use. These tools include: (1) guidelines for harmonized data collection, analysis and presentation, starting with clinical trials; (2) a data collection matrix; (3) a glossary and ontology of terms enabling mapping of the case definitions to several main disease codes used in electronic health care databases; and (4) an online tool for automated case classification of text descriptions of AEFI. These tools will also be published in one of the special Vaccine issues on GAIA when ready and available on the GAIA and Brighton Collaboration websites. With such a solid scientific foundation, we can be optimistic that the field of maternal immunizations, especially our understanding of its safety, can now advance. In an era of increased vaccine hesitancy and skepticism [19], GAIA may serve as a model for how one realm of immunizations can move from the fear of the unknown to a sound evidence base.
